# Polymer encapsulated microcavity optomechanical magnetometer

**DOI:** 10.1038/s41598-017-08875-1

**Published:** 2017-08-21

**Authors:** Jiangang Zhu, Guangming Zhao, Igor Savukov, Lan Yang

**Affiliations:** 1 Los Alamos National Lab, Los Alamos, NM 87544 USA; 2 Department of Electrical and Systems Engineering, Washington University, St. Louis, MO 63130 USA

## Abstract

We demonstrate a magnetometer using polymer encapsulated whispering-gallery-mode microcavity actuated by a micro-magnet. The magnetic field induces force on the micro-magnet causing deformation in the polymer around the microcavity. Subsequently the microcavity detects the change in the refractive index of the polymer resulted from the deformation. This magnetometer works in the frequency range of hertz-to-kilohertz range and achieves a sensitivity of 880 pT/Hz^1/2^ at 200 Hz in a micro-scale sensor volume. Polymer encapsulation of the magnetometer and fiber optical connection ensures environmental robustness and practicality of the sensor.

## Introduction

Ultra-sensitive magnetometers are indispensable for many applications such as magnetic resonance imaging and geological surveying. Magnetometers take many forms such as superconducting quantum interference devices (SQUIDs), Hall-sensors, nitrogen-vacancy (NV) center magnetometers and spin-exchange relaxation-free (SERF) magnetometers^1–4^. In the past, the field was dominated by SQUIDs, which achieve sensitivity of fT/Hz^1/2^ level. However SQUIDs suffer from major drawbacks of requiring cryogenic infrastructure and maintenance, which makes them hardly suitable for miniaturization applications. Hall sensors use Lorentz force on current to detect magnetic field. They are popular choices for applications because of their low cost and flexibility, but their sensitivity is limited to nT/Hz^1/2^ range due to electronic noise. Currently, NV-center diamond magnetometers are gaining popularity and achieved pT/Hz^1/2^ sensitivity at room temperature. They have unique advantage of nanoscale volume, but are usually constrained by the use of complex optical readout and the need for a microwave system. When used at larger scales, they are less competitive than other sensors. Finally, a cm-size SERF magnetometer achieved record sensitivity of 100 aT/Hz^1/2^, but when the size of the atomic cell is reduced to a few mm, the sensitivity becomes only a few fT, and sub-mm SERF magnetometers were not constructed. It is important to note that SERF operation requires accurate zeroing of magnetic fields and expensive shielding.

Recently, optical magnetostrictive magnetometers are gaining attention for their high sensitivity, flexible size, non-cryogenic working condition and simple optical readout. Examples include microscale magnetostrictive microcantilevers and fiber interferometers with sensitivity of fT/Hz^1/2^ and sizes of several centimeters^5, 6^. The use of optical cavity for the readout of the deformation induced by magnetic field has major advantage over other methods because of strongly resonance-amplified optical signal with possibility for highly localized measurement^7, 8^. In the previous demonstration with optical cavities, peak sensitivities of 131 pT/Hz^1/2^ and 200 pT/Hz^1/2^ were reported in the 100 kHz to MHz range with a centimeter scale cavity and a microcavity, respectively^8, 9^. On the other hand, the low frequency range (hertz-to-kilohertz) magnetic detection is crucial to many applications. The previous work on optomechanical microcavity magnetometer achieved a sensitivity of 150 nT/Hz^1/2^ over the range of 2 Hz to 1 kHz^8^. These demonstrations showed very promising results but were performed in a finely controlled environment on an optical bench with an advanced readout system, thereby limiting their practical use.

In this work, we demonstrate a robust polymer-packaged opto-mechanical magnetometer with microcavity readout. We use microtoroid cavities as sensors because of their ultra-high Q factors and small mode volumes^10–13^. By integrating a micro-magnet in soft polymer material near the optical microcavity, our magnetometer works in the hertz-to-kilohertz frequency range and achieves a greatly improved sensitivity of 880 pT/Hz^1/2^ at 200 Hz with a dynamic range of 4 decades. Our magnetometer employs a direct optical readout method of the mechanical signal without the use of any advanced signal processing techniques, and the system realization only requires essential external components such as a laser and a photo-receiver.

## Device fabrication and principle

We fabricated microtoroidal microcavities with major diameters of about 120–150 microns and minor diameters of about 10 microns^10^. The microtoroids have intrinsic Q factors ~5 × 10^7^. A tapered fiber is used to couple laser light into the microtoroid. The tapered fiber is aligned with the microtoroid using a nanopositioning stage with 10 nm resolution. Light from a tunable laser source (NewFocus Velocity Series) is sent through the tapered fiber and is monitored at the output end by a photoreceiver (Newport 1801FC). The wavelength of the input light is linearly scanned by applying a triangular signal to the laser controller so the transmission spectrum of the system can be monitored continuously^11^. An optical resonance is continuously monitored in the polymer encapsulation process. Low index UV curable polymer is used to cover the microtoroid and tapered fiber, and subsequent curing of the polymer makes the microtoroid-fiber system a robust package^14–16^. Because the polymer fixes the gap between the fiber taper and the microtoroid, the coupling between the taper and cavity is constant. After packaging, the Q factors of the microcavities decrease to about 10^6^, which is still very high, sufficient for many sensing applications. The package is demonstrated to work outside the optical bench and could be used as a portable sensing platform^14–16^.

**Figure 1:**
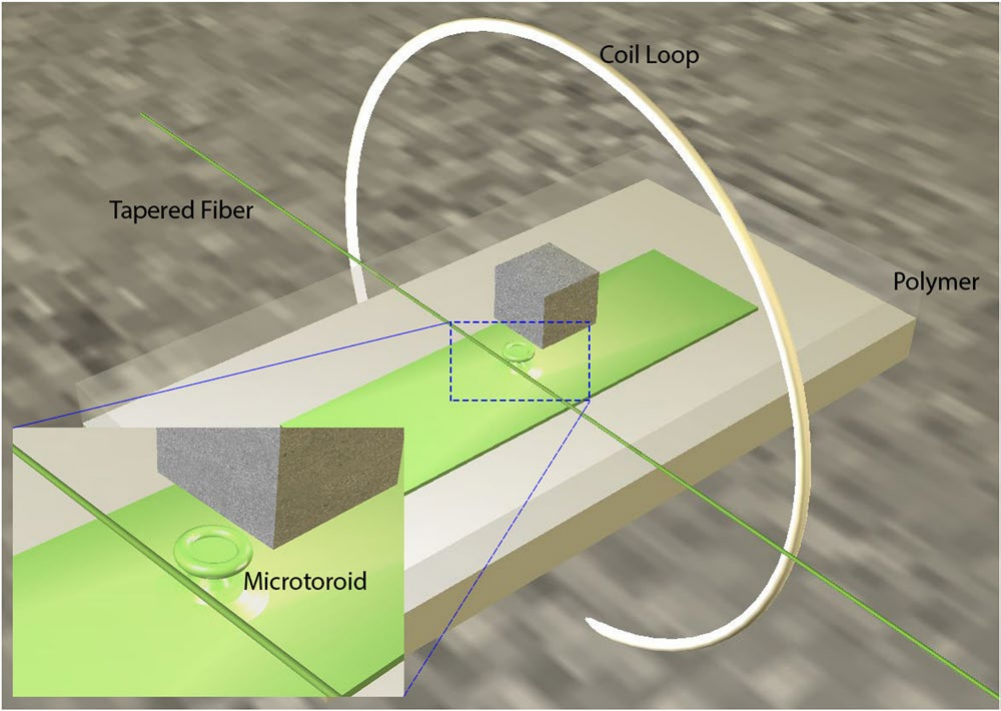
Illustration of the experimental setup of the microcavity magnetometer. The chip (green) with microtoroids and a tapered fiber (green) are encapsulated under polymer. The micro-magnet (cube) is glued above the microtoroid. Inset shows a magnified view of the microtoroid and the tapered fiber.

To enable magnetic sensing applications, the polymer encapsulation process is carefully performed so the polymer layer above the microcavity is very thin. We estimated this thickness to be about 200 to 500 microns. Note that since the microtoroid is a suspended structure (Fig. 1), the polymer thickness above the chip substrate (outside the top of microtoroid) can be much thicker, which increases the strength of the package. The thin layer of polymer above the microcavity allows pressure to be detected by the microcavity. When the polymer is under compression, local deformation occurs which causes the density and refractive index of polymer to change. Since the microcavity is made of silica glass, its density and refractive index change is significantly smaller than the soft polymer. Since a small part (evanescent tail) of the WGM is within the polymer material^14^, the refractive index change of the polymer will alter the resonance condition and cause the optical resonance wavelength to shift. This shift can be sensitively detected by monitoring the resonance transmission. To facilitate this principle and detect magnetic field, we simply glued a small Neodymium magnet in the area above the microcavity using UV glue. We tried magnets with different sizes of 2 mm and 500 microns. The 500 micron magnet was obtained by crushing a bigger magnet, so the shape of the magnet is not regular. Smaller magnets than 500 microns were found to work poorly possibly because of loss of magnetization resulting in diminishing magnetic force on the magnet. If the polymer above the microcavity is thinner or the magnet can be placed closer to the microcavity, smaller magnets may be used.

The magnetic field was generated by a circular two-turn 4-cm diameter coil. The coil was positioned in such a way that the magnet was in its center (Fig. 1) and a sinusoidal current signal of known frequency and amplitude was applied to the coil and monitored in real time with an oscilloscope. To maximize the response, the position and orientation of the micro magnet was carefully adjusted on the surface of polymer with respect to the micro-resonator before glue fixation. In this configuration, the magnet is at the center of the coil loop and only experiences torque, thus, the maximal response could be achieved when the magnetic momentum of the micro magnet is aligned to the plane of the coil and perpendicular to the substrate. The magnet experiences an oscillating torque which exerts local pressure on the polymer (Fig. 1). For chosen mechanical properties of the system (polymer stiffness, magnet mass), the magnet-polymer system is more sensitive to frequencies in the hertz to kilo-hertz range.

## Results

**Figure 2:**
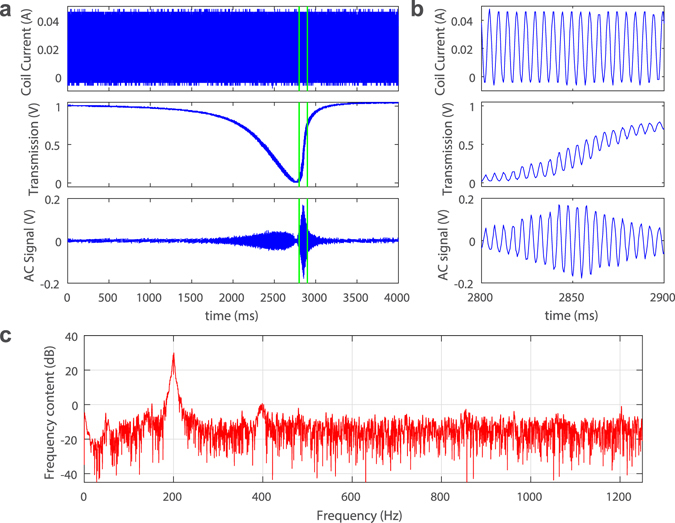
Experimental results. (**a**) Response of the optical resonance to the varying magnetic fields. Upper panel: driving current of the magnetic coil; Middle panel: optical resonance mode showing thermal heating effect caused by the probe laser (triangular lineshape), and overlaying oscillating signal in response to the magnetic field; Lower panel: time varying components of the optical resonance signal in the middle panel. (**b**) Detailed view of the data between the two green lines in (**a**). (**c**) Power spectrum of the signal in the lower panel of (**a**) between time 2 s and 3 s.

To demonstrate the principle of magnetic sensing, first we located a high Q resonance mode (Q ~ 10^6^) and obtained its transmission spectrum (Fig. 2a). The scanning speed of the tunable laser was set to a slower speed (4.8 GHz/s) to visualize the fine details on the spectrum. The laser power was set to be about several hundred microwatts, as measured by the photoreceiver. Due to optical heating effect of the probe laser, an asymmetric triangular lineshape instead of a Lorentzian shape occurred in the transmission spectrum^17, 18^. The slower scanning speed leads to greater thermal buildup and enhances thermal effect. When an oscillation current of 50 mA (peak-to-peak) and frequency of 200 Hz was applied to the coil, we observed fine oscillation patterns overlaying on the otherwise smooth transmission spectrum. This is due to the shift of the resonance frequency by the polymer refractive index change induced by the magnetic force. To better visualize this oscillating signal on transmission, the smooth component of the transmission was removed, and we obtained the pure oscillating signal in the lower panel of Fig. 2a. As expected, the amplitude of this signal is proportional to the slope of the resonance curve, which confirms that the origin of this signal is the resonance frequency shift. The asymmetric lineshape of the transmission results in higher response amplitude in the uprising slope of the resonance. Therefore, higher optical power could result in stronger thermal effect, steeper uprising slope, and hence enhancement in the overall sensitivity, if sensing is performed on the unstable side of the spectrum (steeper slope side)^19^. Note that the frequency of the input signal is within hertz to kilo-hertz range, and the signal period is much larger than the thermal relaxation time (thermal time constant) of the microcavity; thus thermal heating should not have significant effect on the bandwith of the sensor^19^. Figure 2b shows the detailed signal in Fig. 2a between the two vertical green lines. The clearly observed waveform indicated a high signal-to-noise ratio (SNR). We further analyzed the frequency components of the signal in Fig. 2a between time of 2 s and 3 s (Fig. 2c), which reveals a peak at 200 Hz corresponding to the applied oscillating magnetic field. Note that there is a slight second harmonic peak at 400 Hz, which is the result of the nonlinear resonance lineshape.

**Figure 3:**
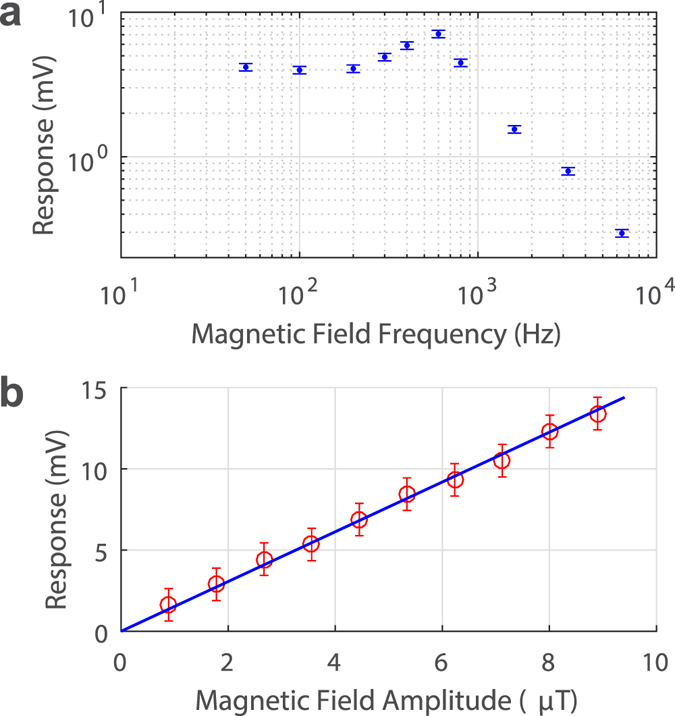
Frequency and amplitude response. (**a**) Frequency response and (**b**) amplitude response of the microcavity magnetometer.

In the following experiments, we fixed (no feedback locking) the probe laser wavelength on the steeper side of the resonance slope where sensitivity is the highest (between the two green lines in Fig. 2a). We also set the data acquisition in AC mode to reject the DC signal component to continuously monitor only the oscillating transmission signal. Next, we varied the frequency of the coil current for a constant current amplitude of 25 mA to obtain the magnetometer frequency response. The result is shown in Fig. 3a. A peak appeared at 600 Hz, which is likely due to the mechanical resonance of the magnet-polymer system. A smaller size magnet would have higher characteristic mechanical resonance frequency, but could be less responsive to magnetic field than a larger magnet. The response starts to fall off rapidly above 1 kHz. Here the bandwidth is limited by the mechanical aspect of the system. The sensor performs very well in the Hz-to-kHz range which should enable many important applications.

Next we kept the frequency of the magnetic field at constant 200 Hz and varied the amplitude of the coil current. The sensor response at different magnetic field amplitudes is plotted in Fig. 3b. A linear relationship between the input and output is observed up to about 9 μT, limited by the maximum output of our current source. From strictly linear response in the studied range and linearity of mechanical properties of the system, we expect the linear response even in much wider range. In experiment, we placed a small Neodymium magnet near the package and saw very large shift in resonance without damaging the package. Therefore, we estimate the maximum detectable field to be above 10 mT. However, the sensor response may not be linear when field become larger. In this study, due to the capability of our current driver and the coil setup, the linearity response curve (Fig. 3b) only goes to 9 μT.

**Figure 4:**
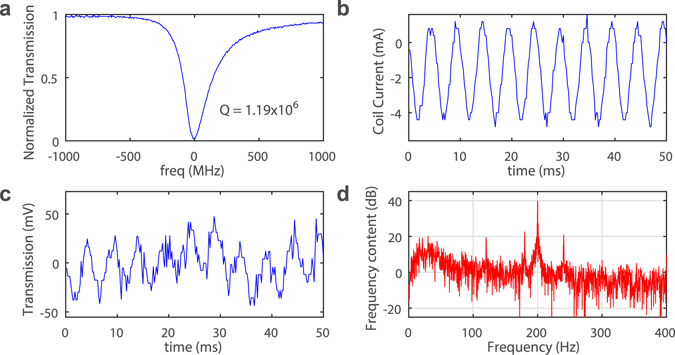
Experimental results for sensitivity. (**a**) The optical resonance mode used for testing shows a Q factor of 1.19 × 10^6^. Laser wavelength is 980 nm. (**b**) Oscillating Coil current with amplitude of 2.5 mA. (**c**) Measured response from the microcavity magnetometer. (**d**) Power spectrum of the magnetometer signal showing a peak at 200 Hz with SNR of about 39 dB. Time domain data acquiring window is 1 s.

In our final experiments, we tested the detection limit of our magnetometer. We used an optical resonance mode with Q factor of 1.19 × 10^6^ which was achieved at critical coupling (Fig. 4a). The coil is single turn to reduce magnetic field and the oscillating coil current is set to amplitude of 2.5 mA and frequency of 200 Hz. The probe laser wavelength was finely adjusted and fixed on the resonance slope to maximize the output response. Figure 4b and c show the coil current signal and sensor output signal, respectively. The magnetic field amplitude at the center of the coil is calculated to be 78.5 nT. The power spectrum of the sensor output signal (Fig. 4c) is plotted in Fig. 4d, which shows a peak at 200 Hz with a signal-noise-ratio (SNR) of 39 dB. This corresponds to a sensitivity of 880 pT/Hz^1/2^. Therefore, this magnetometer should cover at least 4 decades of dynamic range (880 pT to 9 μT).

## Conclusion

In summary, we demonstrated a WGM microcavity magnetometer in a compact and robust package that provides mechanical and optical stability. Polymer packaging eliminates the need for careful optical alignment usually required for microcavity sensors. The experiments were carried out in an open lab environment without magnetic shielding and complex signal acquiring techniques. We demonstrated a high sensitivity of 880 pT/Hz^1/2^ and a 4-decade dynamic range. The dynamic range can be extended by locking the probe laser on the resonance. The combination of the performance, robustness and optical fiber connectivity of the magnetometer should enable some unique applications. The sensor performance is mostly limited by the mechanical response and optical Q factors. This magnetometer can be potentially improved by placing the micro-magnet closer to the microcavity, using different materials for the microcavity and encapsulating polymer, or optimizing the mechanical configuration of the microcavity. Similar realization should be possible with other types of optical microcavity and waveguides, especially optical components made of soft materials.
